# Understanding Job Satisfaction and Occupational Stressors of Distinctive Roles in Zoos and Aquariums

**DOI:** 10.3390/ani13122018

**Published:** 2023-06-17

**Authors:** Sabrina Brando, Patrícia Rachinas-Lopes, Vinícius Donisete Lima Rodrigues Goulart, Lynette A. Hart

**Affiliations:** 1 AnimalConcepts, P.O. Box 378, 03725 Teulada, Spain; 2 Division of Psychology, Faculty of Natural Sciences, University of Stirling, Stirling FK9 4LA, UK; 3 MARE—Marine and Environmental Sciences Centre/ARTNET—Aquatic Research Network, Ispa—Instituto Universitário de Ciências Psicológicas, Sociais e da Vida, Rua Jardim do Tabaco, 34, 1149-041 Lisboa, Portugal; 4 Transportation Research and Environmental Modelling Lab—TREM, Institute of Geosciences, Universidade Federal de Minas Gerais, Belo Horizonte 31270-901, MG, Brazil; 5 Department of Population Health & Reproduction, School of Veterinary Medicine, University of California, Davis, 3207 VM3B, Davis, CA 95616, USA

**Keywords:** human wellbeing, animal wellbeing, animal welfare, hedonic, eudaimonic

## Abstract

**Simple Summary:**

The present study aimed to identify common joys and stressors experienced by zoo professionals working in a variety of roles ranging from junior animal care staff to curators. While many of those who care for non-human animals professionally identify their work to be purposeful, meaningful, and intrinsic to their calling and values, there can be downfalls to their chosen careers. Through a survey of 311 zoo and aquarium professionals, the present study identified common themes about their lack of ability to feel empowered to do their best for animal welfare. We identified a link between staff welfare and perceptions of animal welfare, highlighting areas that organisations can target to improve the ability of their staff to care for animals by taking better care of their people by reducing stressors.

**Abstract:**

For professionals caring for humans or non-human animals, many joys are to be found in working towards what an individual believes to be their calling, especially as they contribute to purposeful, meaningful work consistent with and intrinsic to their own values and beliefs. However, there can be downfalls. Empathic strain, conflict between co-workers, dissatisfaction with upper management, lack of opportunities to make positive changes, limited or no access to level and experience-appropriate professional development, and other stressors are all risks carried by organisations concerned with animal welfare. In the present study, a survey on job satisfaction and workplace stressors was completed by 311 zoo and aquarium professionals working in a range of roles from junior animal care staff to curator. Respondent profiles were created using Multiple Correspondence Analysis (MCA) and four distinct clusters were identified through Hierarchical Clustering on Principal Components (HCPC), highlighting common themes in different levels of experience and in job roles regarding stressors, satisfaction, and feelings about their work and workplaces. Overall, many zoo professionals were concerned with lacking the ability to feel empowered to do their best for animal welfare, and they described a link between the staff welfare and their perceptions of the welfare of the animals they cared for. Through identifying and understanding where organisations can better support their staff it is possible to target and reduce the number of common stressors faced by zoo professionals, leading to increased staff retention, higher job satisfaction, and an improved ability to perform at their best for animal welfare.

## 1. Introduction

For those with a passion for animals, working with and caring for domestic and exotic species is a lifelong dream. Being an animal care professional—the term we will use to describe those working in zoos and aquariums (henceforth zoos)—is often considered more than an occupation. Working in zoos has often been described as a “calling”, based on a person’s deep-seated passion and commitment to achieve excellence in animal care that yields positive welfare and contributing to conservation and education outcomes [1]. However, the field does not typically offer high economic status, extra perks, or opportunities for advancement. Interviews with zookeepers have found that while they feel their work gives them meaning and identity, it also comes with an acceptance of obligation, sacrifice, and unbending duty [1]. While specific numbers are missing, it is estimated that there are approximately 10,000 zoos around the world, of which an estimated 1000 are accredited by zoo and aquarium associations. Staff numbers per facility range from as few as 5 to over 250 care professionals depending on the country, size, and budget of the facility (personal observations, Brando). These estimates do not include wildlife centres, sanctuaries, rescue centres, and other facilities caring for wild and domesticated animals that may be open to the public. While exact numbers globally are unknown, the authors have contacted zoo and aquarium associations to try to state an approximate number, and it would be safe to say that tens of thousands of people are employed in animal care roles in zoos and aquariums globally. Understanding the joys and sorrows of caring for animals maintained in zoos is key to creating a culture of care at the individual, team, leadership, and organisational levels. The zoo community also includes educators, grounds staff, volunteers, and others who care, see, or communicate about the animals on a regular basis; however, they were not the focus of this study.

As with the other animal caregiving contexts, the wellbeing of zoo animals and human caregivers is highly interconnected. To work towards a culture of care, we must first understand the physical and psychological experiences of animal care professionals. Who and what do animal care professionals care for and care about? What was their motivation to get into this profession? Why do people stay in this profession despite difficult conditions such as dealing with work overload, poor animal welfare, or weather-related strains, such as cold and rain and often poor financial incentives? Next to care and passion for animals, what are the other things that make this work meaningful, enjoyable, and interesting? What do individuals, leaders, and organisations do to support and celebrate their staff and animals, such as access to continued professional development, adequate budgets for animal care and wellbeing, and health care in certain parts of the world? What different phases do people move through over the long term during their careers, and how do they experience these phases? What do people who are working in these professions need? Many questions arise when we want to better understand the human wellbeing domains and work-personal integration in zoos or aquariums, and not all will be answered in this paper.

Despite the vast numbers of people employed for such work, there is little information available on the effects of caring for animals in zoos, including occupational stresses and hazards, and the opportunities and joys experienced in and related to the zoo animal care profession. This small community includes caregivers, curators, veterinarians, directors, and others working closely with and around animals. They experience both positive [2,3,4] and more challenging [5] relationships with their animals. A qualitative study documented zookeepers viewing their jobs as a calling, yet they also felt exploited or overworked [1]. Employers may take advantage of zookeepers’ passion and willingness to work despite the challenges of their role (e.g., weather, workload, and limited budgets), and may not feel that they need to reward such employees or provide incentives. Work as a calling can be associated with greater work meaning, job satisfaction, and life satisfaction, but it also carries a potential dark side with the risk of exploitation from employers [6]; for example, not showing the salary prior to application, low wages, and relying on people’s passion for animals and the environment. 

Possible positive occupational aspects of being an animal care professional, such as feeling empowered to act for animals and species or conservation of the planet, connecting visitors to the natural world, and contributing to research, are not well-studied. Negative occupational stresses and hazards, such as moral stress, empathic strain, depression, burnout, grief and loss, and the unique contributing factors, are also poorly understood in zoos today, but these stressors are well-documented in other caregiving contexts, including small animal practice [7,8,9,10], laboratory animal settings [10,11,12,13], and animal shelters [14]. Animal caregiving is well-known to be stressful and joyful; however, the challenges and opportunities posed by the profession vary between contexts, disciplines, and facilities, and therefore, each should be researched. To understand stressors inherent in animal care-related professions, some background information, guidance, and inspiration are relevant from the aforementioned shelter, veterinary, and laboratory contexts. Inherent negative stressors have been identified across these other contexts of animal care, and these studies have highlighted the types of stressors encountered and the terminologies used. A broader meaning and significance of their work are reported by Bunderson and Thompson [1], but further insights on positive opportunities and perceptions are lacking. Fakkema writes, “Those of us who work on behalf of and who dedicate our lives to animals go through four phases in our career evolution.” He describes that we are all unique and all have our own personal stories, but we go through a similar process in our careers. The Four Phases of Career Evolution [15], respectively, are Honeymoon, Depression, Anger, and Resilience.

This paper explores caregiver wellbeing in different roles, ranging from junior zoo professionals up to management positions, and identifies areas in which caregivers in zoos or aquariums both experience and perceive the different challenges and opportunities presented as their careers evolve. Organisations and people can then better serve the animals in their care and further other goals, such as conservation, education, and research, with resilience, joy, purpose, and integrity, and do so in a sustainable manner. When human well-being improves, so too does the well-being of the animals in their care.

## 2. Materials and Methods

### 2.1. Study Design

A customised survey was developed with the aim of gaining insights as to what animal care professionals in zoos and aquariums are experiencing and perceiving the range of positive to negative aspects of their job, including their job satisfaction, negative stressors on the job, and how they monitored and perceived animal welfare.

The questionnaire was designed to target the population of professionals currently working in zoos. Questions were developed by the authors based on their consolidated experiences in the field, combined with adapted survey questions described by Boivin et al. [16] and addressing relevant indicators on the research topic and following the design by Krosnick [17]. A question indicating the sex or gender of respondents was deliberately omitted as this survey did not have any other questions that would dive deeper into differences based on gender but which will be investigated in ongoing work.

The finalised survey comprised 26 questions, including choices from a dropdown menu listing different countries; selecting all options that applied; questions based on a Likert scale with 5 options to select (always, often, sometimes, seldom, and never); and open-ended questions. An invitation to the survey was emailed to all members of AnimalConcepts’ mailing list, consisting of animal care staff, trainers, veterinarians, and other professionals working with animals, e.g., zoos and sanctuaries. The groups were identified based on the target audience of people working in zoos and aquariums. The invitations were sent several times and posted on different zoo and aquarium Facebook groups, AnimalConcepts’ Instagram account, LinkedIn, and Twitter. It is not possible to calculate a response rate from the list or social media as this question was not included in the survey. AnimalConcepts is a company working globally in the space of animal, human, and planetary wellbeing, collaborating with zoos, aquariums, sanctuaries, wildlife centres, universities, research facilities, and companion and farm animal organisations in support of our collective goals of optimal wellbeing for animals and the people who care for them, and conservation, engagement, and research.

**Figure 1:**
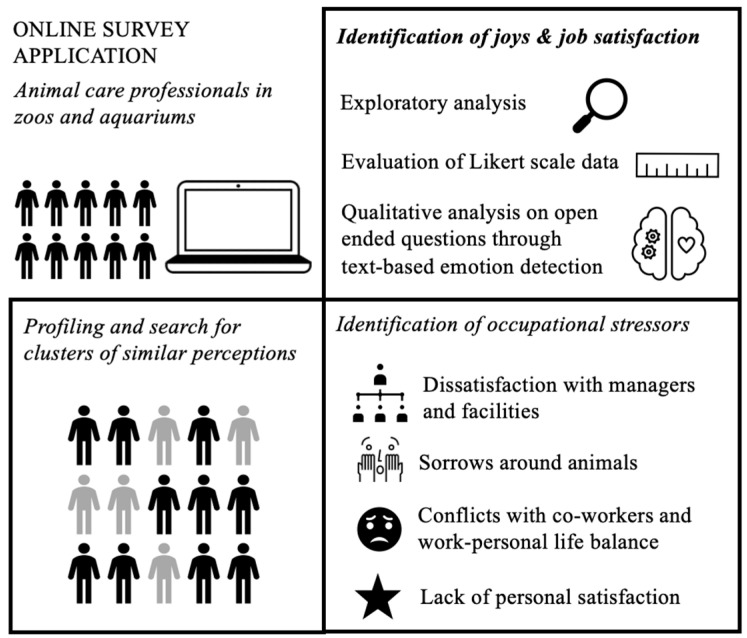
Stages of research and approaches employed for profiling participants: exploratory analysis, Multiple Correspondence Analysis and Hierarchical Clustering on Principal Components, identifying job satisfaction (Sentiment Analysis and Likert scale data), and investigating occupational stressors (ANOVA comparing identified occupational stressors).

The survey structure is intended to evaluate occupational stressors and classify them by following comprehensive categories: Overall personal satisfaction; Management dissatisfaction; Conflict with co-workers and work–life balance; and Sorrows around animals (see Figure 1). Once the occupational stressors categories were described by the questionnaire data, the participants were grouped in clusters to investigate the effects of individual features (e.g., time working in the field) on personal job satisfaction and attitudes. The data was also explored qualitatively through the employment of sentiment analysis using natural language processing methodologies. Through the development of this work, it will be possible to answer the following research questions: Is there a trend in occupational stressors for workers in zoos? How is the job perceived (e.g., calling); is it possible to find groups with different perceptions among the respondents?

### 2.2. Statistical Analyses

Data were processed and treated for consistency before any further analysis. In the case of ambiguous answers to job descriptions, the most reasonable information was selected. As an example, where some participants selected both junior and senior animal caregiver as their job description, the higher job description was selected, which in the given example would be the senior animal caregiver. 

Since not all participants fully completed the questionnaire, those with a minimum of 50% completion were included for further statistical analysis. Exploratory data analysis was employed to investigate the overall features and distribution of the data, using absolute frequencies, percentages, averages, standard deviations, and graphical analyses. Survey questions based on the Likert scale were processed by converting available options into a numerical scale. The alternatives were Never, Seldom, Sometimes, Often, and Always. Given that questions have a negative (e.g., At my job, I feel:—Sad) or positive valence (e.g., At my job, I feel:—Happy), the selection was converted into numbers from one to five. In the case of positive questions, the scale was Never (1), Seldom (2), Sometimes (3), Often (4), and Always (5); for negative questions, the levels were reversed for Never (5), Seldom (4), Sometimes (3), Often (2), and Always (1). The score was analysed by averages and standard deviations and tested for normality. For multiple selection questions, where more than one option could be selected, the frequency of each alternative was used to describe the subject of the question. The questionnaire was built with four main topics to investigate job satisfaction and occupational stressors. Overall personal satisfaction, regarding the description of perceptions about the role at the zoo; Management dissatisfaction, regarding the recognition from the employer and facilities; Conflict with co-workers and work-personal life balance, regarding the working routine and interaction with the team; and Sorrows around animals, regarding the suffering from the psychological attachment with the animals cared for. These four comprehensive categories were compared using an ANOVA followed by Tukey’s post hoc test with the average of Likert scores from each related question (please see Supplementary Material). 

A chi-square analysis was employed to investigate the distribution of the years of experience reported and the proportion of answers considering the job role as a calling, career, or role. A multiple correspondence analysis (MCA) was used to identify the principal components for aggregating participants in groups, followed by a Hierarchical Clustering on Principal Components (HCPC) to investigate clusters formed by the respondents. MCA allows the analysis of patterns of relationships between participants by employing nominal variables, such as survey responses. HCPC uses the matrix produced by the MCA to find individuals with a close relationship based on survey answers [18]. Therefore, it is possible to compare patterns of responses from each group identified. The clusters identified were tested using a Kruskal–Wallis for comparing the pattern of responses from Likert scores. Sentiment analysis was employed to identify the sentiment-associated responses linked to each open-ended question. Sentiments and emotions were identified by using Natural Language processing. The text from respondents was evaluated by matching words with an emotion lexicon, which identifies each word with an emotion (e.g., positive or negative) and a sentiment (Joy, Sadness, Anger, Fear, Anticipation, Surprise, Disgust, and Trust) according to the Plutchik model [18]. 

Statistical analyses were performed in the R language and environment for statistical computing (R core team 2021). MCA and HCPC were conducted using the package “FactoMineR” and FactoShiny [17,18]. Missing data were treated using the package “missMDA” before running the analyses [19]. Sentiment analyses were performed using the r package “syuzhet” [20,21,22] with words categorised based on the “NRC Emotion Lexicon”. Figures were constructed using the package ggplot2 [23] and data manipulation using tidyverse [24].

## 3. Results

A total of 311 respondents replied to the survey with mean progress toward completion of 52.2%. The total number of 92 participants completing the entire survey represented a drop-off rate of 70.42%. Regarding job titles, which can be manifold, e.g., being the director and the veterinarian, 99 participants were junior animal caregivers, and 138 were senior animal caregivers, 10 were veterinarians, 39 were curators, and 23 were zoological directors. Two participants did not report their job titles. Participants were experienced professionals regarding wellbeing in the zoo environment. The participants’ origins were mostly from zoos in the United Kingdom and Northern Ireland (*n =* 75) and the United States of America (*n =* 74), together representing 47.91% of the total sample size (*n =* 311). Thus, the results do not represent a wide geographical distribution. Given the high number of incomplete form responses, we considered a minimum of 50% completion for further statistical analysis as described below (*n =* 162).

Regarding the duration-of-experience classes (i.e., I have worked in the zoo/aquarium domain for…), it was observed that most professionals ranged from 1 to 15 years of experience. A total of 24 participants had less than 1 year of experience, 83 participants had from 1 up to 5 years of experience, 78 participants had 5 up to 10 years, 43 had participants 10 up to 15 years, 29 participants had 15–20 years, and 53 participants had more than 20 years of experience. One participant did not report the duration of their experience. The distribution among classes’ duration-of-experience was not equally distributed; some classes were more represented (χ^2^ = 19.036; DF = 5; *p* = 0.0019). The number of more experienced respondents is highlighted, with 65% of participants having more than 5 years of experience in the zoo/aquarium domain.

Most respondents considered life to be more balanced towards work (60.49%, *n =* 98), while 17.9% (*n =* 29) considered the work–life balance “mostly even”; a total of 21 (12.96%) consider the work–life balance “problematic”; the remaining participants believe the work–life balance “Satisfactory” (6.79%, *n =* 11) and only 3 “balanced more toward life”. 

The predominant perception was that the profession of working at a zoo is a calling (61.11%, *n =* 99), while 34.57% (*n =* 56) considered it as a career and only 4.32% (*n =* 7) as a regular job. The proportion of respondents considering the role as a calling was significantly higher (χ^2^ = 78.4815; DF = 2; *p* < 0.0001). Overall, professionals were happy with their chosen profession, with 50.28% answering never having regrets. Therefore, the participants agreed that despite the problems commonly faced at work, their chosen job carried a high vocational meaning.

The main stressor found by the Likert scores “When I leave work I: Think about work while at home”, with an average score of 1.92 ± 0.86. In this case, where the question has a negative valence, low average Likert scores represent that participants think about work at home frequently. Participants reported that: I worry about animals when I leave work ($\bar{X}$ = 2.40 ± 0.97). Another source of dissatisfaction is the lack of opportunities for recognition ($\bar{X}$ = 2.02 ± 1.17). Feelings of frustration ($\bar{X}$ = 2.48 ± 0.87), stress ($\bar{X}$ = 2.49 ± 1), lack of safety ($\bar{X}$ = 2.50 ± 1.23), overwork ($\bar{X}$ = 2.54 ± 1.13), and worn out ($\bar{X}$ = 2.65 ± 1.10) were reported as frequent. These are the most frequent factors impacting job satisfaction in zoos.

**Figure 2:**
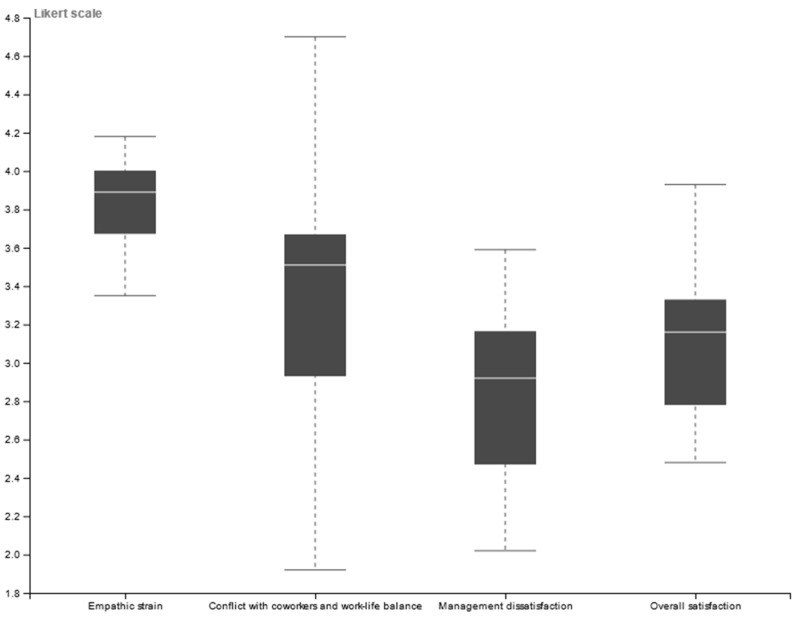
Boxplot with categories of occupational stressors demonstrating the impact on job satisfaction. Data is shown on median scores of Likert scales. Low scores indicate less satisfaction.

The “overall personal satisfaction”, “Management dissatisfaction”, “Conflict with co-workers and work-personal life balance”, and “Empathic strain” categories were compared to evaluate differences in occupational stressors. We found significant differences among the occupational stressors (F = 11.6731; DF = 3; *p* < 0.0001). The lowest score (i.e., potential stressor) is found for “management dissatisfaction” ($\bar{X}$ = 2.81 ± 0.50) which is significantly different from the other categories. A post hoc Tukey test highlights significant differences between “Empathic strain” (*p* < 0.0001) and “Conflict with co-workers and work–life balance (*p* < 0.0195)”. A significant difference was also found between “Empathic strain” and “Overall satisfaction” (*p* < 0.0002), where the overall satisfaction had lower scores, therefore a source of stress (Figure 2). Therefore, the main occupational stressor affecting job satisfaction is management. It is important to note that low Likert scores represent worse conditions (Supplementary Table S1).

### 3.1. Participants’ Profiles

**Figure 3:**
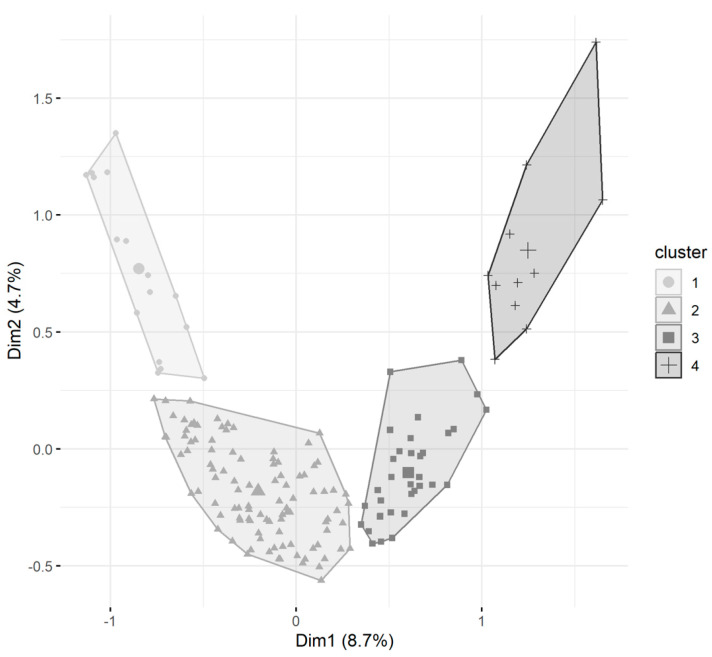
Factor map showing the distribution of participants with varying durations of experience and attitudes to their work. Dim1 (First Dimension) and Dim2 (Second Dimension) represent the transformation of variables and reduction in the multivariate dataset into dimensions through the variation on principal components.

The participants’ profiles were identified by a multiple correspondence analysis (MCA) by selecting the most representative answered questions. Once the dimensions were defined, a Hierarchical Clustering on Principal Components (HCPC) was performed to detect the clusters relative to each participant. Each cluster was characterised, revealing the most relevant variables for groups. The categorical variables from the survey questions were transformed into continuous variables represented by the dimensions obtained from the multivariate analysis (MCA). The clusters were identified by numbers, and the principal dimensions were used to plot the respondents (see Figure 3 and Supplementary Material Figure S1. 

**Figure 4:**
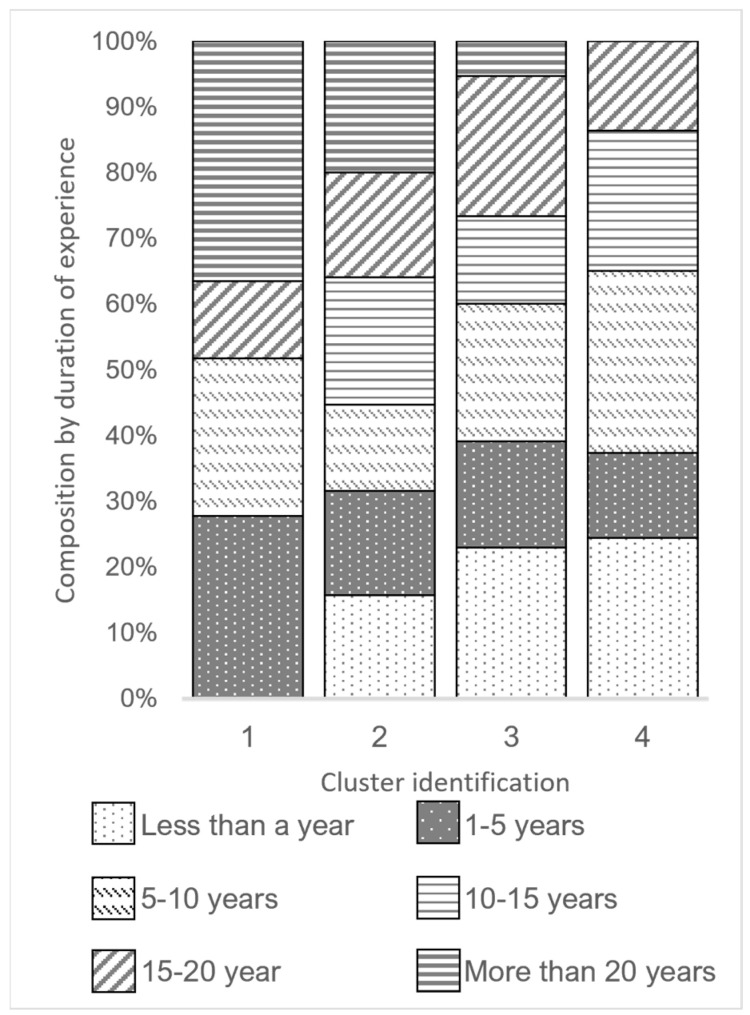
Cluster composition by participants’ durations of experience, showing clusters 1 to 4, with a decreasing number of experienced professionals, and roughly arranged by levels of satisfaction in the workplace.

A total of four clusters were identified by the HCPC (Figure 3). The clusters were characterised by similar responses in the questionnaire. Most are on a Likert scale from Never, Seldom, Sometimes, Often, and Always. The four clusters can be roughly described by the duration of experience of the participants (Figure 4). 

The first cluster (*n =* 16, see Appendix A Table A1) describes a group including professionals with a variable duration of work (1–5 years up to more than 20 years) and experienced professionals (senior animal caregiver, curator, and zoological director). In the most positive cluster, members of this group felt respected at work and were rarely ignored. They felt safe and happy with their chosen job, without regret. They received good support from their departments. Regarding the animals, this group considered that the animals were in good facilities and had a good quality of life. This group also felt that there should be opportunities to talk about losing an animal.

The second cluster (*n =* 101, see Appendix A Table A2) is formed by experienced professionals (e.g., curators) with a calling to make a difference for animals. Fairly dissatisfied, they seldom felt respected and recognised, often being frustrated and feeling ignored. Their sentiments had a negative valence toward sadness and anger. They varied in enthusiasm for the daily routine and were indifferent to acknowledgements. Mostly, they were not satisfied with their institutions.

The third cluster (*n =* 34, see Appendix A Table A3) is formed by professionals varying in their durations of working in a zoo setting, but mostly they were experienced professionals. Additionally, dissatisfied, they had a pronounced frustration and felt frequently ignored. Sadness and angry feelings were common, but they sometimes felt satisfied with the position. The workload impacted their welfare. Usually, they did not have institutional support for professional development. Social relationships were a problem at work, with poor interactions among the staff. This group described a poor daily routine and dreaded the day ahead. 

The last cluster (*n =* 11, see Appendix A Table A4) is characterised by inexperienced professionals that did not feel respected; they also were not satisfied with their position. They received good support from the zoo but did not feel valued. They had problems relating to the staff. They believed that the animals were not provided with good welfare standards. 

### 3.2. Sentiment Analysis

**Figure 5:**
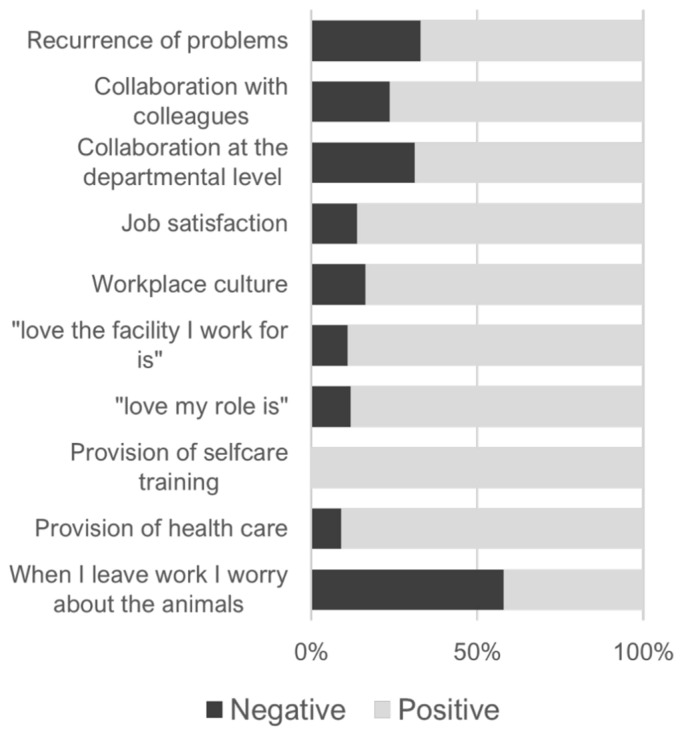
Sentiments associated with the job satisfaction of workers in zoos were identified from sentiment analysis of open-ended questions. Recurrence of problems (“I run into the same problems all the time”); Collaboration with colleagues (“At my job I collaborate well with colleagues to get things done for animals”); Collaboration at the departmental level (”Would you say good collaboration is at departmental and/or management level?”); Job Satisfaction (“JOB SATISFACTION—At my job I”); Workplace culture (“I think the culture in the workplace could be improved by”); “love the facility I work for is” (“What is going to help me—love the facility I work for is”); ”love my role is” (“What is going to help me—love my role is”); Provision of selfcare training (“The facility I work at provides/has:—Selfcare training (e.g., resilience training, compassion awareness)” Provision of health care (“The facility I work at provides/has:—Health care“)); When I leave work I worry about the animals (“When I leave work I:—Worry about the animals (you can add comments on your worries if you want)”).

The sentiment analysis from the commentary section indicates that the predominant emotion, according to the NRC emotion lexicon (National Research Council Canada), is “Trust” (475 words identified) (see Supplementary Material Figure S2, and the prevalent sentiment for the whole questionnaire was positive (79.97%) with 587 words under the positive category and 147 words with negative valence (Figure 5).

The most negative sentiment was found for the question, “When I leave work I: Worry about the animals”. This question describes concerns retained when leaving work by the participant, demonstrating the impacts and the closed relationship with the cared animal. The main concern was identified by words, such as “ill”, “sick”, “death”, and “forgotten”, revealing attention to the health status of the animal. The impossibility of continuing with the care of their animal(s) after working hours is illustrated by the comment, “I want to change [general welfare levels] for the better but can’t and there’s nothing I can do and I’ll have to see it all again tomorrow”. 

The most answered question was “I think the culture in the workplace could be improved by “…”, having been described in greater detail by the participants with a positive sentiment demonstrated by 179 positive words and 160 for the emotion Trust. Better communication and management were frequently cited as an area for improvement. Ideas for improvement of workplace conditions were highlighted by descriptions such as “professional”, “cooperation”, “respect”, “development”, “progression”, and “passionate”. For instance, the following comments illustrate the culture in the workplace: “Decreasing workload so staff can participate in more conservation projects and professional development activities”; “creating opportunities for teambuilding and collaboration within the facility and the greater zoological/conservation community”; and “More accountability as well as team-based goals”. One respondent indicated that they felt there was a need for spaces to share “more of a voice to the dissatisfaction many feel, but don’t have a good opportunity to express.”

It is worth mentioning the presence of comments and suggestions for improvement which indicate unsatisfactory and aggressive feelings across zoo professionals, such as “Paying us more”; “People being kinder to themselves and each other”; “Educating keepers [on] animal behaviour and welfare, ethics)”; “Respect, less selfishness”; “A complete change in a bullying management team, replaced by a team that actually knew about captive animal welfare and how to manage staff”; and “Some egos being burst”.

## 4. Discussion

The present study assessed caregiver wellbeing in zoo professionals across different roles, ranging from junior to management positions, by identifying areas in which caregivers in zoos experience and perceive different challenges and opportunities throughout their careers. As in animal welfare, when considering human wellbeing, we need to think about the cumulative effects of work-related stressors through stressors. By understanding the compounded effects of the joys and the sorrows experienced day in and day out by caregivers, we may be able to provide support and opportunities to improve and promote positive wellbeing and high levels of job satisfaction. 

### 4.1. Participant Profiles

Among all participants, 61.11% indicated that their job was a calling and that they were happy with the chosen profession, not showing regrets and agreeing that despite problems commonly faced at work, the chosen job was appropriate with high vocational meaning. They also reported that they were happy with the state of the animals housed in their institution, reflecting a consensus on appropriate care and successful animal welfare actions. Through all job categories, participants indicated “Always” and “Often” in more than 50% of their total answers that they see animals who are well, who trust the staff, are in positive animal welfare states, live in appropriate social groups, and play or hang out with the public, reflecting a consensus on appropriate care and successful animal welfare actions. It is notable that participants that had negative perceptions about the job also seemed to have negative perceptions about the animals’ welfare. This aligns with results reported by Riggio et al. [25] that a more positive perception of Brambell’s Freedoms for zoo canids was associated with higher job satisfaction. Maybe the participants have negative perceptions about their jobs because they see animals with a negative state of wellbeing.

Some inexperienced professionals (less than 1 year of experience) did not feel respected and were not satisfied with the position and although they received good support from the zoo, they did not feel valued. They also had problems relating with other members of staff and believed that the animals were not provided good welfare. Some also reported that they sometimes encountered animals who engaged in stereotypic behaviour, were doing poorly, lived in substandard facilities, spent most of their time in the back of the display area, and experienced a low quality of life.

From the observed clusters, it is possible to conclude that the duration of the working experience at a zoo is positively related with the job satisfaction. Some professionals with a variable time of work (1–5 years up to more than 20 years) and experienced professionals (senior animal caregiver, curator, and zoological director) felt respected at work and were rarely ignored, felt perfectly safe and were happy with the chosen job without regret, received good support from their department, and considered that the animals were in good facilities and had a good quality of life. The “honeymoon” phase (for less experienced professionals) and resilience phase (for more experienced professionals), as described by Fakkema [15] may account for these results. Regarding euthanasia, all participants of this survey reported that there should be an opportunity to talk about losing an animal. There may be too little attention to this part of an animal’s life; attention to all aspects around euthanasia should be reviewed, consulting with all.

Years of experience in working with zoos characterise cluster formation progressively. This would somewhat reflect people leaving their workplaces if dissatisfied, leaving the more satisfied workers with longer durations in the workplace. Some experienced professionals (e.g., curators) who were identified as having a calling to make a difference for animals seldom felt respected and recognised, instead often being frustrated, and feeling ignored. Their sentiment has a negative valence toward sadness and anger; they varied in their enthusiasm for the daily routine and were indifferent regarding whether they received acknowledgements. Usually, these experienced upper-level professionals did not have institutional support for their professional development. From an equity and inclusion perspective, it seemed unfair that appropriate opportunities commensurate with their job level and experience were not being offered to all staff. Animal care and welfare is a rapidly expanding and evolving field, and many zoo professionals are interested in advancing their careers through opportunities for personal development [26,27]. Some of these professionals felt they could affect structural changes, but also that they had fewer opportunities to make a difference for the animals at their facility. This points to a perception within upper-level management of being more distantly involved with the animals they are working with. Many curators and zoological directors grow into these positions over a period of many years, where work shifts from practical animal care to more administrative tasks, reporting, scheduling, and meetings, which could account for their perception of the meaningfulness of their work being reduced. Sometimes people grow into these jobs not necessarily because of their interest in the specific role but because of their work experience, duration, and seniority in the facility, and the financial opportunities available when in higher management positions. Considering these professionals may have more opportunities to affect structural change within their facilities compared to less experienced staff, a reframing of their perceptions regarding their roles might help individuals in upper management roles to find more meaning in their work and empower their ability to make changes for animals. Supporting middle managers to care for and encourage their teams and furthering other organisational goals, will be key to keeping these professionals interested and committed and may include being effective umbrella carriers [28] and advancing their managerial capabilities [29]. 

While responses of “Always”, “Often”, “Seldom”, and “Never”, are important, the answer of “Sometimes” can also be vital to consider. A large number of people indicating that some of the time they feel happy or frustrated, accompanied by the frequency of emotional highs being lower and the frequency of emotional lows being higher, may indicate a perpetual or chronic state of suboptimal wellbeing. Alternatively, it could indicate that people have found an equilibrium that they are satisfied with or resigned to what seems to be a repetitive or similar situation most of the time. This is particularly important to consider when people have been in the field for a long time—perhaps they are passing through stages of depression and anger within their career and will perhaps then leave the job or find a way that works for them and move into the resilience phase.

### 4.2. Sentiment Analysis

Most of the participants, independently of their job descriptions, indicated that the predominant sentiments about collaborations with colleagues were positive. Such results demonstrate that professionalism toward the animals and the aim of caring for them prevail over other negative sentiments, such as, for example, those associated with problems in the workplace. Collaboration toward caring for animals also indicated a relationship of trust between colleagues. This is significant because, while the job was not always easy, with feelings of frustration and being overwhelmed, most people stayed in—and were content with—their job overall. Today, we often talk about work–life integration rather than a work–life balance: meaning that the personal life away from work and life at work are intrinsically connected and intertwined. Particularly in work, when it is seen as a calling and meaningful to the individual, the pleasurable (hedonistic) aspects of work are important; however, the flourishing and purposeful (eudaimonic) aspects of work may outweigh the pleasurable ones (for a review see, e.g., [30]). People in a profession which they feel is meaningful see and feel that their work has a purpose; they care for and about the animals, and they care about other goals, such as protecting biodiversity and the planet.

However, it is important to consider the potential negative impact on staff wellbeing that their connection and passion towards animals might bring. Most prominently, the results indicated that many animal care staff found it difficult to “switch off” from their jobs when they returned home. The predominant sentiment towards animal contact during out-of-work hours was sadness with participants, independently of their job description, indicating that they think and worry about the animals at their workplace when they are not there. Compassion fatigue (today there is a shift in terminology to empathic strain) is a well-documented phenomenon for those who work and forge bonds with animals [7,8] and support networks between staff within the facility are essential in combating the pain that can come with worrying about animals out-of-hours. The connection to the animals is like an invisible string that cannot be severed, and it seems likely they would not want it any other way. This is reflected in the results for all three groups indicating that they “Seldom” regret choosing their jobs.

Regarding the provision of free health care for staff for the different participant’s job description, anticipation was the main sentiment—probably because this is dependent on the country. In countries where people have free access to a social health care system, this is of less or no concern, while in other countries, it is a valued and important contractual benefit. It is also worth acknowledging that health care can take the form of provisions for both physical and psychological support, both on location at the organisation, and access to external professionals. Many animal care roles are physically demanding, with the potential for both acute and chronic injury and workplace stress can arise from the mental and social demands of the profession. Regardless, there were many differences in answers regarding what the institution provides to the staff during working hours and what may help them to love their roles, love their facility, and improve the workplace. The main sentiment expressed by the participants was trust: a feeling of acceptance that they are safe in their workplace in all definitions of the term and that it is possible to be open, connected, and allied with those they work with [31]. Sometimes facilities provide staff areas, clothing, a certain number of free tickets for friends and family, and/or other incentives. Going beyond yoga and a staff appreciation day, which are both excellent activities and efforts, it will be fundamental to understand what truly matters and what is meaningful and important as perceived by the staff themselves. Considering all the different answers given by participants, it will be interesting for future research to uncover what participants consider to be good working conditions. 

Potential sorrows from their jobs may include feeling frustrated, overwhelmed, or inadequately served in the workplace, and other components may contribute to joys from their jobs, such as feeling empowered and being an actor in animal care, promoter of positive animal welfare, and contributing to conservation efforts. Interventions found to be successful in other animal caregiving contexts may also be found to be effective in zoos or perhaps could be adapted for zoos [8]; by understanding where communication, support systems, or the facilities themselves either fall short or are supportive to care staff, we can more effectively target negative stressors, i.e., preventing burnout, depression, empathic strain, and other potential negative outcomes, such as an increased risk of poor mental health [32]. This can point the way toward providing support and opportunities to improve and promote positive wellbeing, high levels of job satisfaction, and healthy work-personal life integration for those working in zoos.

Understanding these perceptions and feelings, and the factors that may contribute to suboptimal staff wellbeing on a chronic level, will allow employers within the field to make team-, leadership-, and institutional-level changes that promote staff happiness, retention, and flourishing in the long term. As indicated from research in other professions, happy staff stay for longer in their jobs and reduce turnover [33,34], may progress into advanced roles through mentoring programs within their facility [33], and are better able to forge beneficial human–animal bonds with the animals they work with and thus improve animal welfare [35,36]. The results of the present study indicate that despite having a great passion for their jobs, staff at all experience levels express concerns that they do not always have appropriate opportunities for professional development and that they are not always able to do what they believe to be best for the animals. They report that their issues with workplace rapport can cause frustration and sadness that contributes to stress and unhappiness in their roles, otherwise seen as a calling in life. By uncovering and tackling the causes of these negative feelings, facilities concerned with animal and staff welfare can make actionable changes to improve both. This may be through staff training opportunities, team building, offering different incentives, or other means. 

## 5. Limitations

Questions in the first section of the survey revolved around what people can do for animals together with their colleagues, and the second section of the survey was on how organisations celebrate the animals, i.e., what they do for animals. The second section had a drop in the number of responses. The responses that were given for both sections were consistent with each other in terms of the overall emotional valence of responses. Survey fatigue may have performed a role in the drop-off, or possibly respondents felt that the questions about their individual or team behaviours seemed similar to how the organisation was facilitating animal care and welfare goals, which four people expressed in the notes. For this pilot study, we did not enquire on the size of the organisation, which may also have an effect, and did not have information on whether the organisation the participant worked for at the time of the survey was accredited or not. Many of these questions were left unanswered. Even though this survey was filled out anonymously, people may have felt uncomfortable answering certain questions, might not have known the answers, or may have had survey fatigue at the end of this somewhat long survey. 

This survey did not address the detailed specifics of meaningful work nor the effect of human wellbeing on animal wellbeing. Much more could be explored regarding the duties performed in each job role and how these are perceived by the various groups, including different genders, which is part of ongoing work. Finally, a deeper dive into the questions related to “do for the animals” is part of an ongoing study which aims to shed more light on the relationship between support and care for animals and its effects on human wellbeing.

## 6. Conclusions

Overall, while there was variation in the specific stressors and joys experienced by animal care staff at different levels, respondents in all clusters and indeed across the profession were united by their passion for their jobs and desired to do right by the animals in their care. However, not all respondents felt supported or celebrated in their roles, with discrepancies between the feelings of less experienced staff and those in higher-level management positions. When this has a knock-on effect on animal welfare, the negative feelings of staff are not only detrimental to the organisation’s ability to retain and prevent negative wellbeing in staff but also to their own missions of animal welfare. 

Through developing a better understanding of the needs, feelings, and desires of their staff, it is possible to uncover where communication, support systems, or the facilities themselves fall short in ensuring that care staff have a safe and open environment to perform well in their roles. Armed with this knowledge, organisations can better target negative stressors in the workplace that may contribute to burnout, depression, empathy fatigue, and other negative outcomes. Organisations and people are then able to serve animals and further their other goals, such as conservation, education, and research, with resilience, joy, purpose, and integrity, and can do so in a sustainable manner. When human wellbeing improves, so does the wellbeing of the animals in their care. Understanding the joys and sorrows of caring for captive animals in zoos is key to creating a culture of care, both from an individual and an organisational level. 

More research is needed on the cumulative exposure of infrequent (marked as “Sometimes” and “Seldom”) events, such as suffering individuals, seeing animals afraid of some people, awaiting transport alone for a long time in the back of the display area for an educational program, stereotyping animals, caring for ill and ageing animals, or euthanising a loved animal. These all can be a strain on animal care professionals, and the compounded impact of repeated events carries a real risk of compassion fatigue [8]. Perceptions of animal welfare can be altered by the feelings brought by the animal care professionals, particularly the ones that are working close to the animals through their own physical and psychological states. The present study functions as a starting point for organisations to acknowledge their possible downfalls regarding staff welfare and take steps towards change and for future research which explores the specifics of individual stressors for zoo professionals in more detail. 

## Data Availability

Please contact the corresponding author for data availability.

## Supplementary Materials

The following supporting information can be downloaded at: https://www.mdpi.com/article/10.3390/ani13122018/s1, Table S1: Scores obtained for Likert scale data presenting the average ($\;\bar{x}$) and standard deviation (±) of identified occupational stressors. Figure S1: Cluster dendrogram from Hierarchical Clustering on Principal Components classifying participants in the survey on job satisfaction and occupational stressors. Dashed boxes highlight the identified cluster solution and participants identified by numbers on the edge of the dendrogram. Figure S2: Proportions of emotions detected by sentiment analysis applied on open-ended questions anserwed by animal care professionals in zoological institutions.

## Appendix A

**Table A1:** Characterisation of cluster number one is based on Hierarchical Clustering on Principal Components: professionals with varying durations of experience and positive attitudes to the workplace.

Cluster	Topic	Question	Agreement
1	When I leave work I:	Feel satisfied that I am effectively contributing to important goals.	Always
	How satisfied are you with	Communication.	Often
		Support from your management.	Often
		Attention to promoting optimal animal welfare.	Always
	At my job, I feel	Respected.	Always
		Helpless.	Never
		Indifferent.	Never
		Stressed.	Sometimes
		I feel perfectly 100% safe right now.	Always
		Happy.	Always
		Trapped.	Never
	When I go to work I	Look forward to caring for the animals.	Always
		Feel happy I have chosen this job.	Always
		Regret having chosen this job.	Never
		Dread the day ahead of me.	Never

**Table A2:** Characterisation of cluster number two is based on Hierarchical Clustering on Principal Components: fairly dissatisfied experienced professionals.

Cluster	Topic	Question	Agreement
2	When I leave work I	Worry about the animals.	Sometimes
	How satisfied are you with	Support from your management.	Sometimes
	At my job, I feel	Satisfied.	Often
		Worn out.	Sometimes
		Valued.	Often
		Ignored.	Sometimes
	When I go to work I	Look forward to a new day.	Often

**Table A3:** Characterisation of cluster number three is based on Hierarchical Clustering on Principal Components: dissatisfied professionals varying in experience.

Cluster	Topic	Question	Agreement
3	How satisfied are you with	Departmental financing.	Seldom
	At my job, I see and experience	Animals who live in great social groups.	Often
		Animals who spend a lot of time relaxing, playing, and in positive states.	Often
	At my job, I feel	Helpless.	Often
		Empowered.	Seldom
		Valued.	Sometimes
		Heard.	Sometimes
		Happy.	Sometimes
	My employer/facility celebrates me through	Providing lunch.	Never
		Providing funds to attend a conference or equivalent on a fair rotation basis (not paying for these expenses yourself).	Never
		Providing opportunities to participate in volunteering programs of choice.	Never
		Team bonding exercises or events.	Never
	When I go to work, I	Feel annoyed/worried/scared at facing the same problems.	Often
		Feel happy I have chosen this job.	Sometimes

**Table A4:** Characterisation of cluster number four is based on Hierarchical Clustering of Principal Components: dissatisfied and inexperienced professionals.

Cluster	Topic	Question	Agreement
4	When I leave work, I	Worry about the animals (you can add comments on your worries if you want).	Always
		Feel dissatisfied.	Often
	How satisfied are you with	Support from your management.	Never
		Annual review process.	Never
	At my job, I feel	Angry.	Often
		Helpless.	Always
		Empowered.	Never
		Stressed.	Always
		Worn out.	Always
		Overworked.	Always
		Recognised.	Never
		Frustrated.	Always
	My employer/facility celebrates me through	Providing days off to attend a conference or equivalent on a fair rotation basis (not taking your own holidays).	Never
		Providing funds to attend a conference or equivalent on a fair rotation basis (not paying for these expenses yourself).	Never
		Providing opportunities to participate in volunteering programs of choice.	Never
		Team bonding exercises or events.	Never
	When I go to work, I	Look forward to a new day.	Seldom
