# Supplementary material for: Understanding Job Satisfaction and Occupational Stressors of Distinctive Roles in Zoos and Aquariums
Source: Animals (Basel). 2023 Jun 17;13(12):2018. doi: 10.3390/ani13122018 (PMC10295341; doi:10.3390/ani13122018)

# Understanding Job Satisfaction and Occupational Stressors of Distinctive Roles in Zoos and Aquariums

**Sabrina Brando [1,2,*], Patrícia Rachinas-Lopes [1,3], Vinícius Donisete Lima Rodrigues Goulart [4] and Lynette A. Hart [5]**

***Supplementary Table 1****: Scores obtained for Likert scale data presenting the average (x̄) and standard deviation (±) of identified occupational stressors*

| Empathic strain | Mean, SD |
|---|---|
| At my job I see and experience: - Animals for whom it would be more humane to be euthanized | X̄=4.18±0.87 |
| At my job I see and experience: - Animals who are all well most of the time | X̄=4.05±0.74 |
| At my job I see and experience: - Animals who are doing poorly most of the time | X̄=3.90±0.88 |
| At my job I see and experience: - Animals who are waiting to go to another facility and are not in optimal conditions | X̄=4.02±0.93 |
| At my job I see and experience: - Animals who exhibit trust in me and other caregivers | X̄=4.17±0.72 |
| At my job I see and experience: - Animals who have a low quality of life | X̄=3.93±0.97 |
| At my job I see and experience: - Animals who like to play or hang out near the public | X̄=3.35±0.87 |
| At my job I see and experience: - Animals who live in great social groups | X̄=3.77±0.71 |
| At my job I see and experience: - Animals who live in poor social groups | X̄=3.64±0.87 |

| | |
|---|---|
| At my job I see and experience: - Animals who spend a lot of time relaxing. playing and in positive states | $\bar{X}$=3.95±0.61 |
| At my job I see and experience: - Animals who spend all their days in substandard facilities | $\bar{X}$=3.55±1.13 |
| At my job I see and experience: - Animals who spend all their days in the back of house area | $\bar{X}$=3.84±1.11 |
| At my job I see and experience: - Animals who stereotype a large part of the day. and probably the rest of the time too | $\bar{X}$=3.56±1.10 |
| At my job I see and experience: - Traumatized animals (e.g.. animals who are scared most of the time. have high levels of anxiety) | $\bar{X}$=3.88±0.86 |

| Conflict with co-workers and work-life balance | Mean, SD |
| --- | --- |
| When I go to work I : Dread the day ahead of me | X̄=3.64±1.01 |
| When I go to work I  - Feel annoyed/worried/scared about interacting/seeing the team | X̄=3.51±1.10 |
| When I go to work I  - Feel annoyed/worried/scared at facing the same problems | X̄=2.93±1.18 |
| When I go to work I  - Feel happy I have chosen this job | X̄=4.09±0.90 |
| When I go to work I  - Look forward to a new day | X̄=3.64±0.97 |
| When I go to work I : - Look forward to caring for the animals | X̄=4.70±0.59 |
| When I go to work I  - Look forward to seeing the team | X̄=3.67±1.03 |
| When I go to work I  - Regret having chosen this job | X̄=4.14±0.97 |
| When I leave work I: - Feel dissatisfied | X̄=3.13±0.92 |
| When I leave work I: - Feel relaxed and leave work behind | X̄=2.77±1.05 |
| When I leave work I: - Feel satisfied that I am effectively contributing to important goals | X̄=3.35±1.03 |
| When I leave work I: - Think about work while at home | X̄=1.92±0.86 |
| When I leave work I: - Worry about the animals | X̄=2.40±0.97 |

| Management dissatisfaction | Mean, SD |
|---|---|
| How satisfied are you with: - Annual review process | X̄=2.69±1.25 |
| How satisfied are you with: - Attention to promoting optimal animal welfare | X̄=3.31±1.05 |
| How satisfied are you with: - Communication | X̄=2.92±0.88 |
| How satisfied are you with: - Departmental financing | X̄=2.67±1.10 |
| How satisfied are you with: - Support from your department | X̄=3.45±0.98 |
| How satisfied are you with: - Support from your management | X̄=2.95±1.12 |
| My employer/facility celebrates me through: - Good salary (I do not require 2-3 jobs to live comfortably) | X̄=3.03±1.26 |
| My employer/facility celebrates me through: - Good working conditions (e.g.. appropriate clothing. equipment) | X̄=3.59±1.03 |
| My employer/facility celebrates me through: - Opportunities for awards of recognition for exceptional work | X̄=2.02±1.17 |
| My employer/facility celebrates me through: - Providing coffee/tea | X̄=3.30±1.70 |
| My employer/facility celebrates me through: - Providing days off to attend a conference or equivalent on a fair rotation basis (not taking your own holidays) | X̄=2.99±1.36 |
| My employer/facility celebrates me through: - Providing funds to attend a conference or equivalent on a fair rotation basis (not paying for these expenses yourself) | X̄=2.67±1.26 |
| My employer/facility celebrates me through: - Providing lunch | X̄=2.12±1.31 |

| | |
|---|---|
| My employer/facility celebrates me through: - Providing opportunities to participate in volunteering programs of choice | $\bar{X}$=2.27±1.27 |
| My employer/facility celebrates me through: - Team bonding exercises or events | $\bar{X}$=2.14±1.09 |

| Overall satisfaction | Mean, SD |
|---|---|
| At my job I feel: - Angry | X̄=3.16±0.97 |
| At my job I feel: - Anxious | X̄=2.95±1.10 |
| At my job I feel: - Burdened | X̄=3.28±1.09 |
| At my job I feel: - Empowered | X̄=2.92±1.07 |
| At my job I feel: - Frustrated | X̄=2.48±0.87 |
| At my job I feel: - Happy | X̄=3.66±0.84 |
| At my job I feel: - Heard | X̄=2.78±0.92 |
| At my job I feel: - Helpless | X̄=3.25±1.16 |
| At my job I feel: - I feel perfectly 100% safe right now | X̄=2.50±1.23 |
| At my job I feel: - Ignored | X̄=3.16±1.06 |
| At my job I feel: - Indifferent | X̄=3.93±1.04 |
| At my job I feel: - Overwhelmed | X̄=2.94±0.99 |
| At my job I feel: - Overworked | X̄=2.54±1.13 |

| | |
|---|---|
| At my job I feel: - Recognised | X̄=3.06±0.98 |
| At my job I feel: - Respected | X̄=3.38±1.02 |
| At my job I feel: - Sad | X̄=3.33±0.99 |
| At my job I feel: - Satisfied | X̄=3.37±0.89 |
| At my job I feel: - Stressed | X̄=2.49±1.00 |
| At my job I feel: - Trapped | X̄=3.54±1.26 |
| At my job I feel: - Valued | X̄=3.30±0.97 |
| At my job I feel: - Worn out | X̄=2.65±1.10 |

Figure S1: Cluster dendrogram from Hierarchical Clustering on Principal Components classifying participants in the survey on job satisfaction and occupational stressors. Dashed boxes highlight the identified cluster solution and participants identified by numbers on the edge of the dendrogram.

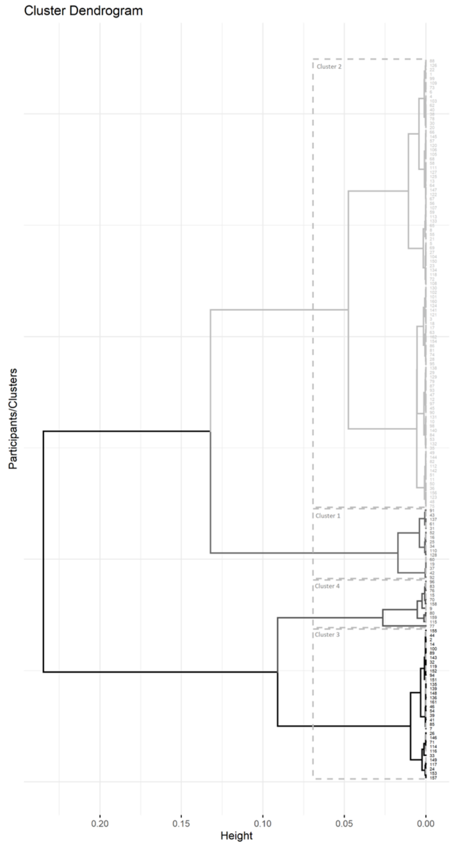

Figure S2: Proportions of emotions detected by sentiment analysis applied on open-ended questions anserwed by animal care professionals in zoological institutions

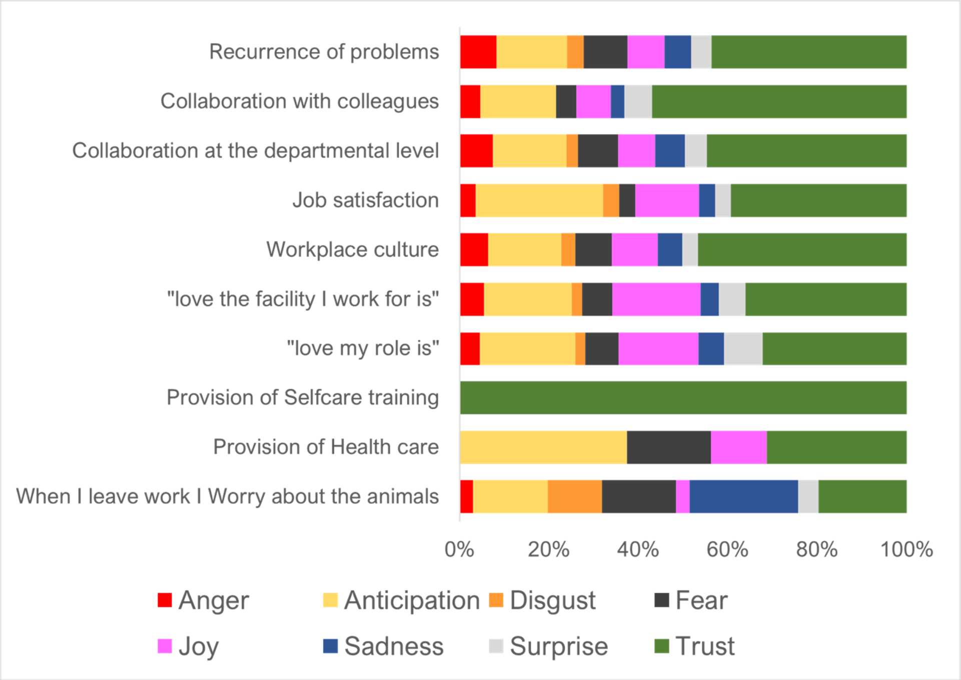

Supplement: Supplementary file 1 [file animals-13-02018-s001.zip › animals-2430329-supplementary.pdf]
